# Characteristics of non-small-cell lung cancer with interstitial pneumonia: variation in cancer location, histopathology, and frequency of postoperative acute exacerbations in interstitial pneumonia

**DOI:** 10.1186/s12890-020-01347-9

**Published:** 2020-11-20

**Authors:** Kazumasa Ogawa, Hironori Uruga, Takeshi Fujii, Sakashi Fujimori, Tadasu Kohno, Atsuko Kurosaki, Kazuma Kishi, Shinji Abe

**Affiliations:** 1 Department of Respiratory Medicine, Respiratory Center, Toranomon Hospital, 2-2-2, Toranomon, Minato-ku, Tokyo, Japan; 2 Okinaka Memorial Institute for Medical Research, 2-2-2, Toranomon, Minato-ku, Tokyo, Japan; 3 Department of Respiratory Medicine, Tokyo Medical University, 6-7-1, Nishi-shinjuku, Shinjuku-ku, Tokyo, Japan; 4 Center for Preventive Medicine, Nomura Hospital, 8-3-6, Shimorenjyaku, Mitaka-shi, Tokyo, Japan; 5 Department of Pathology, Toranomon Hospital, 2-2-2, Toranomon, Minato-ku, Tokyo, Japan; 6 Department of Thoracic Surgery, New Tokyo Hospital, 1271, Wanagaya, Matsudo-shi, Chiba, Japan; 7 Department of Diagnostic Radiology, Japan Anti-Tuberculosis Association, Fukujuji Hospital, 3-1-24, Matsuyama, Kiyose-shi, Tokyo, Japan; 8 Department of Respiratory Medicine, Toho University School of Medicine, 6-11-1, Omori-nishi, Ota-ku, Tokyo, Japan

**Keywords:** Interstitial lung disease, Non–small-cell lung cancer, Video-assisted thoracic surgery, Acute exacerbation, Cancer location, Pulmonary emphysema

## Abstract

**Background:**

Non–small-cell lung cancer (NSCLC) has been reported to develop in patients with interstitial pneumonia (IP); however, clinical, radiological, and pathological features remain to be elucidated.

**Methods:**

We retrieved the records of 120 consecutive NSCLC patients associated with IP who underwent surgery at Toranomon Hospital between June 2011 and May 2017. We classified the patients into three groups according to NSCLC location using high-resolution computed tomography: group A, within a fibrotic shadow and/or at the interface of a fibrotic shadow and normal lung; group B, within emphysematous tissue and/or at the interface of emphysematous tissue and normal lung; and group C, within normal lung. In 64 patients, programmed death ligand-1 (PD-L1) status was assessed with immunohistostaining.

**Results:**

Most of the patients (89; 70%) were classified as group A. This group tended to have squamous cell carcinoma with the usual interstitial pneumonia (UIP). These cancers were located mainly in the lower lobes and seven of the eight postoperative acute exacerbations (pAE) of IP developed in this group. NSCLC in the group B were mainly squamous cell carcinomas located in the upper lobes. No patient with PD-L1 negative was classified into group B. None of the patients in group C showed UIP. and most of the cancers were adenocarcinoma. The frequency of epidermal growth factor receptor mutation-positive NSCLC was the highest in this group.

**Conclusions:**

The three groups each showed characteristic features in terms of tumor location, histopathology, PD-L1 expression, and frequency of pAEof IP.

## Background

For 35 years, lung cancer has been one of the leading causes of cancer-related death in developed countries [1]. Lung cancers have higher incidence rates in patients with interstitial pneumonia (IP) than in those without IP [2–7]. There are various treatment options for lung cancers, including surgical treatment, radiotherapy, chemotherapy, and immunotherapy; however, there is a concern that these may result in the acute exacerbation (AE) of preexisting IP [7–11]. An official joint statement of the American Thoracic Society, European Respiratory Society, Japanese Respiratory Society, and Latin American Thoracic Association (ATS/ERS/JRS/ALAT) [12] weakly recommends steroid therapy for AE of IP; nevertheless, managing AE of IP remains challenging because of the extremely high mortality rate [7, 8, 13–15].

Although there have been reports of the outcomes of surgical treatment for lung cancer patients with IP [8, 16–18], most of these studies were small and retrospective, without long-term outcomes. One of these studies investigated risk factors for the development of postoperative AE of IP [8]. However, as yet there are no established strategies or drugs for preventing AE of IP [7, 8, 19].

The radiological and pathological features of lung cancers associated with IP have not yet been fully elucidated. Some studies have reported that lung cancers with IP tended to develop inside honeycombing in the lungs or at the interface of the honeycombing and normal lung in the lower lobes [7, 20–22], although some lung cancers developed in the upper lobes apart from fibrotic changes [23, 24]. The main pathological type associated with IP is squamous cell carcinoma; in contrast, the main pathological type for patients without IP is adenocarcinoma [7, 25]. The expression of programmed cell death ligand 1(PD-L1) in patients of lung cancer with IP have been also assessed in a few studies and has been mainly reported to be positive [26, 27]. However, studies investigating radiological and pathological features of lung cancers associated with IP have been small and retrospective, and these features require further clarification.

We speculate that lung cancer associated with IP is not a homogeneous disease. Some lung cancers develop within honeycombing in the lungs, whereas others develop within emphysematous tissue in the upper lobes or in normal lung away from fibrotic changes or emphysema. We hypothesized that the features of lung cancers with IP vary according to the location where they develop, as a result of different underlying mechanisms. Additionally, the frequency of postoperative AE of IP may differ according to cancer location. This study aimed to analyze 120 patients with non–small-cell lung cancer (NSCLC) who had IP to verify these hypotheses.

## Methods

This retrospective study was based on the records of patients with NSCLC who underwent surgical treatment at our hospital between June 2011 and May 2017, retrieved from our institutional database. Patients who underwent only surgical biopsy were excluded. Among the 1414 patients with NSCLC, 120 had IP as an underlying condition. These 120 patients were included in our analysis.

The study was approved by the Institutional Review Board of Toranomon Hospital (1329-H/B and 1447). Patient consent was obtained by using informed consent documents with an opt-out process.

In this study, we categorized IP-associated NSCLCs into three groups according to the location of cancer development determined by high-resolution computed tomography (HRCT). We also evaluated the clinical courses, treatment outcomes (including long-term outcomes), and pathological findings in each group.

The patient’s medical charts, laboratory data, pulmonary function test results, HRCT findings, and histopathological findings were reviewed using the most recent results available prior to the surgical treatment. The data and outcomes were followed until July 2019.

In this study, IP diagnoses were established using HRCT. The radiological pattern of IP was classified into the usual interstitial pneumonia (UIP) pattern or non-UIP pattern. The UIP pattern required subpleural, basal predominance, reticular abnormality, and the absence of the non-UIP pattern features on HRCT. Honeycombing or traction bronchiectasis was not essential for the UIP pattern in the present study. The non-UIP pattern required one of the following: upper or mid-lung predominance, peribronchovascular predominance, extensive ground-glass abnormality, profuse micronodules, discrete cysts, diffuse mosaic attenuation, air-trapping, and consolidation in bronchopulmonary segments [12].

The severity of IP was calculated using the Gender–Age–Physiology (GAP) index [28], a multidimensional index and staging system for idiopathic pulmonary fibrosis (IPF). We adapted the GAP index for the patients whose IP was not IPF.

AE of IP were diagnosed using the definition proposed by Collard et al. [29], which requires that all of the following four conditions be met: a previous or concurrent diagnosis of IPF; acute deterioration or the development of dyspnea, typically for less than one month in duration; on CT imaging, new bilateral ground-glass opacity and/or consolidation superimposed on a background of IP consistent with the UIP pattern; and deterioration not fully explained by cardiac failure or fluid overload. In this study, we adapted this definition for patients whose IP was not IPF or whose HRCT images did not exhibit the UIP pattern. Any AE of IP that developed within one month of surgery was considered to be a postoperative AE of IP.

**Fig. 1:**
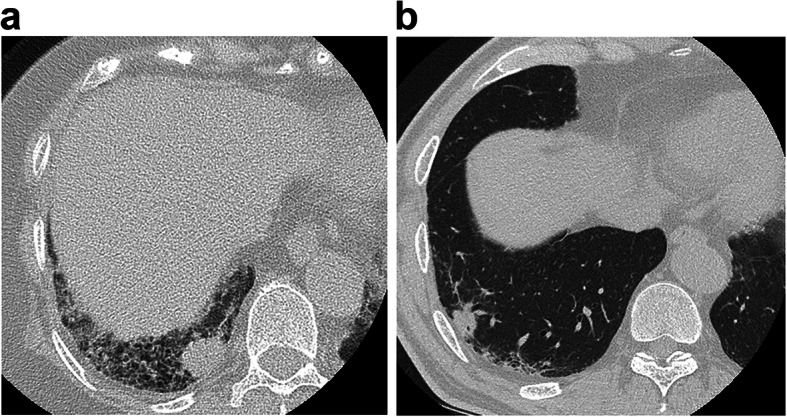
Lung cancer in midst of fibrotic shadow and/or interface of fibrotic shadow and normal lung. These are examples of computed tomography findings for lung cancers in the fibrotic shadow

**Fig. 2:**
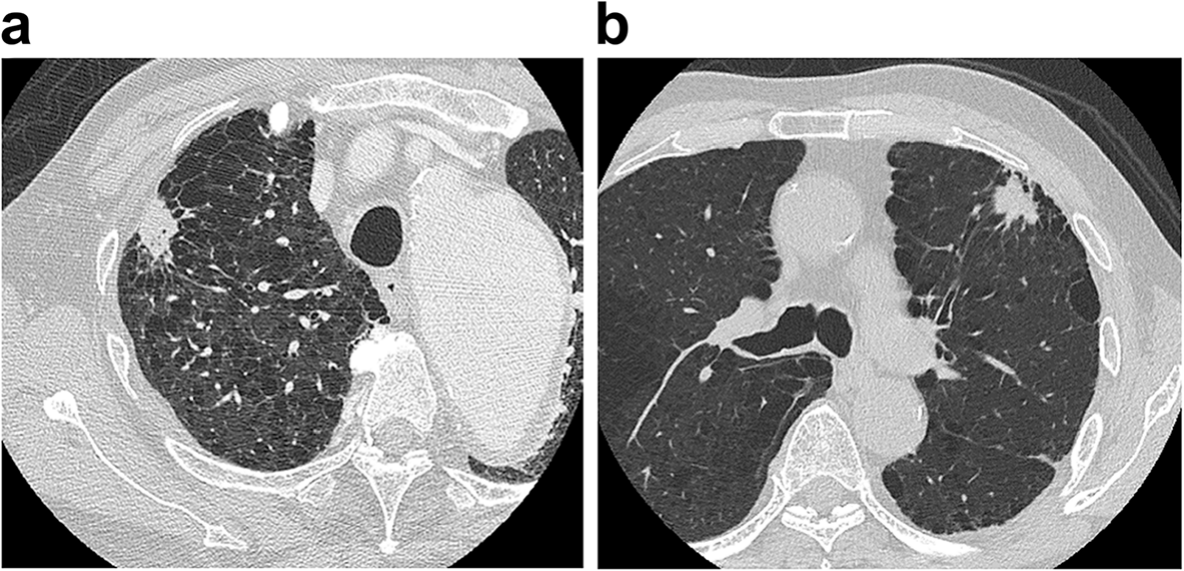
Lung cancer in midst of emphysema and/or interface of emphysema and normal lung. These are examples of computed tomography findings for lung cancers in midst of emphysema

**Fig. 3:**
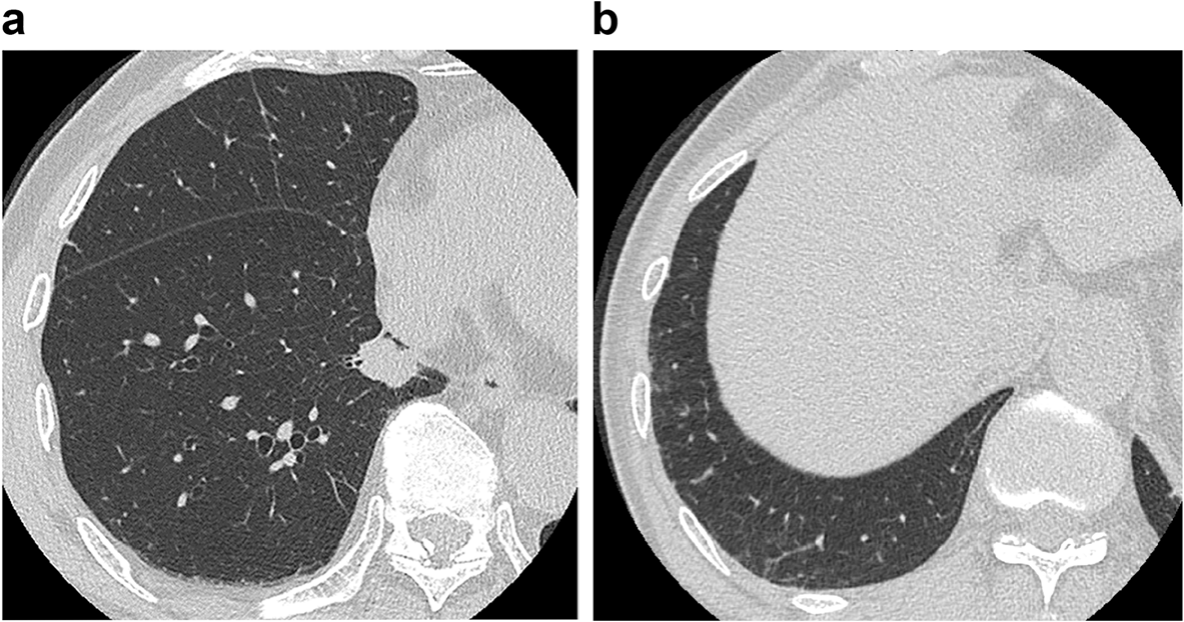
Lung cancer in the normal lung (apart from reticular shadows or emphysema). **a** The example of computed tomography findings in the lung of a patient with lung cancer. **b** This is an image showing rest of the lung of patient with fibrosis in Fig. 3**a.** The image shows mild fibrosis in the right lower lobe; however, it is apart from the lung cancer

We classified lung cancers with IP into three groups according to the location where the cancer developed using HRCT: group A, within a fibrotic shadow and/or at the interface of a fibrotic shadow and normal lung; group B, within emphysematous tissue and/or at the interface of emphysematous tissue and normal lung; and group C, within normal lung, away from reticular shadows or emphysema (Figs. 1, 2, 3).

The histological pattern of the tumor was classified according to the World Health Organization’s Classification of Tumors of the Lung, Pleura, Thymus, and Heart, 4th edition [30], and the cancer was staged according to the American Joint Committee of Cancer’s Cancer Staging Handbook, 7th edition [31].

**Fig. 4:**
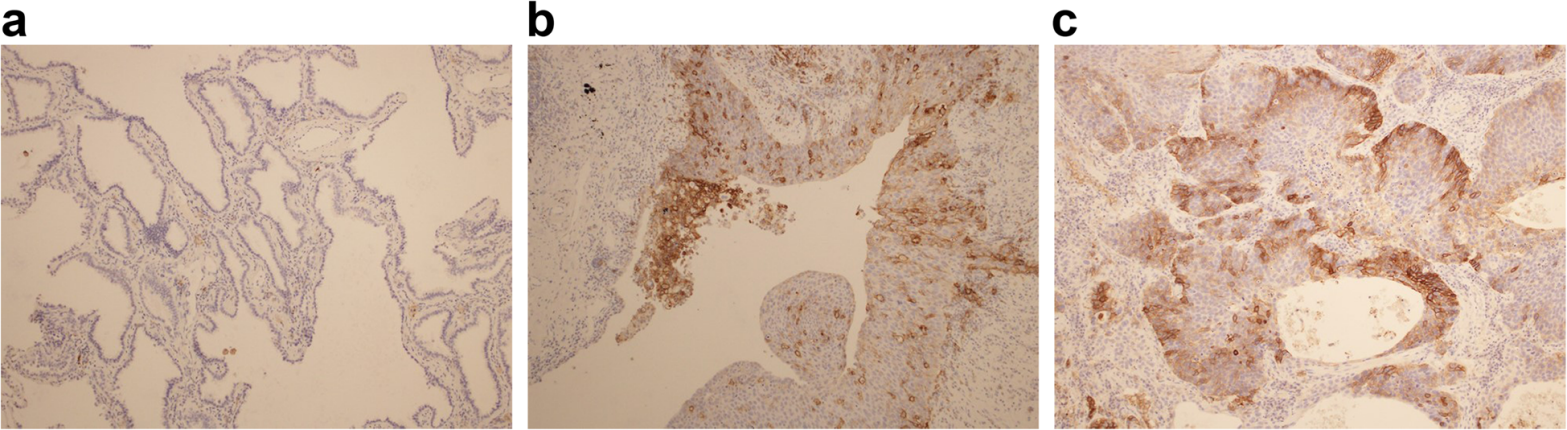
Examples of programmed cell death ligand 1 expression levels assessed using the 22C3 antibody. **a** Tumor proportion score < 1%. **b** Tumor proportion score in the range of 1–49%. **c** Tumor proportion score ≥ 50%

The PD-L1 status of 68 lung cancers in 64 patients who gave written informed consent was assessed by immunohistostaining using 22C3 antibody concentrate on the Dako ASL48 platform. PD-L1 expression was determined according to a tumor proportion score (TPS), which is defined as the percentage of tumor cell-positive membrane staining, and scored independently by one pathologist who specializes in pulmonary pathology and one pulmonologist who specializes in pulmonary pathology; both were blinded to the clinical data. The score was classified into three levels: TPS < 1%, TPS 1–49%, and TPS ≥ 50% (Fig. 4).

The statistical analysis was performed using SPSS version 26.0 (IBM Corp., Armonk, NY). The comparisons of characteristics between the patients with and without the AE of IP were evaluated using the Mann–Whitney U test and chi-square test, as appropriate. Survival time was analyzed with the Kaplan–Meier method. Statistical significance was defined as *p* < 0.05.

## Results

### Clinical results

The clinical and demographic characteristics of the 120 patients (97 men, 23 women; median age, 74 years), including details of their cancers and treatments, are summarized in Table 1. In total there were 128 lung cancers, and eight patients underwent surgical treatment for double de novo lung cancers at once. The median follow-up time was 905 days.

**Table 1:** Patient characteristics

	Characteristic	All patients (*n* = 120)
Age, years		74 (50–90)
Sex	Male/Female	97/23
Etiology of IP	Idiopathic/Other	101/19
Clinical diagnosis of IPF	IPF/Non-IPF	50/70
Surgical approach	VATS/Open surgery	116/4
Pathological staging	Stage Ia/Ib/IIa/IIb/III/IV/undetermined	46/27/16/5 10/2/14
Postoperative adjuvant chemotherapy	Yes/No	8 (6.7%)/112 (93.3%)

Data are presented as number or median (range)

*IP* interstitial pneumonia, *IPF* idiopathic pulmonary fibrosis, *VATS* video-assisted thoracic surgery

All participating patients had IP as an underlying disease, and 101 (84%) of these patients had an idiopathic etiology. Video-assisted thoracic surgery (VATS) alone was used for 116 patients (97%); the other four patients underwent open surgery combined with VATS. Lobectomy was performed in 83 patients, segmentectomy in 23 patients and wedge resection in 14 patients, respectively. Table 1 summarizes the pathological staging of the lung cancer; 46 (38%) of the patients were at stage IA. The undetermined pathological staging was mainly because of wedge resection. Table 2 summarizes the histological types and mutation status of the 128 lung cancers. The most common histological type was squamous cell carcinoma, accounting for 67 (52%) of the cancers. Activating epidermal growth factor receptor mutations were detected in four patients among 111 tested cancers.

**Table 2:** Clinical summary of the 128 lung cancers

		Lung cancers (***n*** = 128)
**Histological type**	**Squamous cell carcinoma** **Adenocarcinoma** **Adenosquamous carcinoma** **Neuroendocrine carcinoma** **Other**	67 (52.3%) 46 (35.9%) 7 (5.5%) 6 (4.7%) 2 (1.6%)
**Mutation status**	**EGFR mutation positive** **ALK translocation positive**	4 (3.1%) 0 (0%)

*EGFR* epidermal growth factor receptor, *ALK* anaplastic lymphoma kinase

Table 3 compares characteristics between the patients who experienced AE of IP and those who did not. Eight patients (7%) experienced postoperative AE of IP. The median time from surgery to the AE was 16.5 days, and the median survival time after surgery of these patients was 176 days. Compared to the other patients, the patients who developed AE of IP tended to have undergone lobectomy (100% vs. 67%, *p* = 0.047) and to have lower percent predicted vital capacity (%VC; 88.0% vs. 100%, *p* = 0.044).

**Table 3:** Comparison of patient characteristics between those with and without acute exacerbation of interstitial pneumonia (IP)

	Characteristic	Without postoperative AE of IP (*n* = 112)	With postoperative AE of IP (*n* = 8)	*P*
Age, years		74.5 (50–90)	71.5 (60–85)	N.S.
Sex	Male	91	6	
	Female	21	2	N.S.
Smoking status	Never	7	0	
	Current or former	105	8	N.S.
Brinkman Index		900 (0–4400)	850 (200–2380)	N.S.
HRCT IP pattern	UIP	52	6	
	Non-UIP	60	2	N.S.
Pulmonary emphysema	Yes	85	7	
	No	27	1	N.S.
Treatment agents for preexisting IP	Steroids	5	0	N.S.
	Antifibrotic agents	1	0	N.S.
Pulmonary function test results	VC (mL)	3290 (1680–5790)	3025 (1920–3910)	N.S.
	% predicted VC	100.0 (55–151)	88.0 (82–106)	0.044
	FVC (mL)	3290 (1560–5790)	2970 (1920–3910)	N.S.
	% predicted FVC	102.1 (56–162)	92.0 (86–114)	N.S.
	% predicted FEV_1.0_	69.9 (41.5–93.1)	77.6 (55.3–82.4)	N.S.
	% predicted DL_co_	74.0 (17–133)	63.0 (48–116)	N.S.
Laboratory data	KL-6 (U/mL)	487.0 (163–5051)	599.0 (233–1386)	N.S.
	SP-D (ng/mL)	109.0 (21.0–542.0)	156.0 (62.1–530.0)	N.S.
	SP-A (ng/mL)	49.8 (17.4–291.6)	67.3 (26.9–143.0)	N.S.
GAP index*		3 (0–6)	2.5 (1–4)	N.S.
Surgical procedure	Lobectomy	75	8	0.047
	Segmentectomy	23	0	N.S.
	Wedge resection	14	0	N.S.

Data are presented as number or median (range)

*IP* interstitial pneumonia, *HRCT* high-resolution computed tomography, *UIP* usual interstitial pneumonia, *VC* vital capacity, *FVC* forced vital capacity, *FEV*_*1.0*_ forced expiratory volume in one second, *DL*_*CO*_ diffusing capacity for carbon monoxide, *KL-6* Krebs von den Lungen-6, *SP-D* surfactant protein D, *SP-A* surfactant protein A, *GAP* Gender–Age–Physiology, *N.S*. not significant

* The GAP index was calculated using a multidimensional index and staging system for idiopathic pulmonary fibrosis [28]

Postoperative lung cancer recurrence was experienced by 29 (24%) of the patients. Best supportive care was the most frequently chosen option for these patients (used for 17 patients, 59%), followed by chemotherapy (10, 34%), and other options (2, 7%). The median survival time from the surgery of the patients with recurrence of lung cancer was 1116 days.

**Fig. 5:**
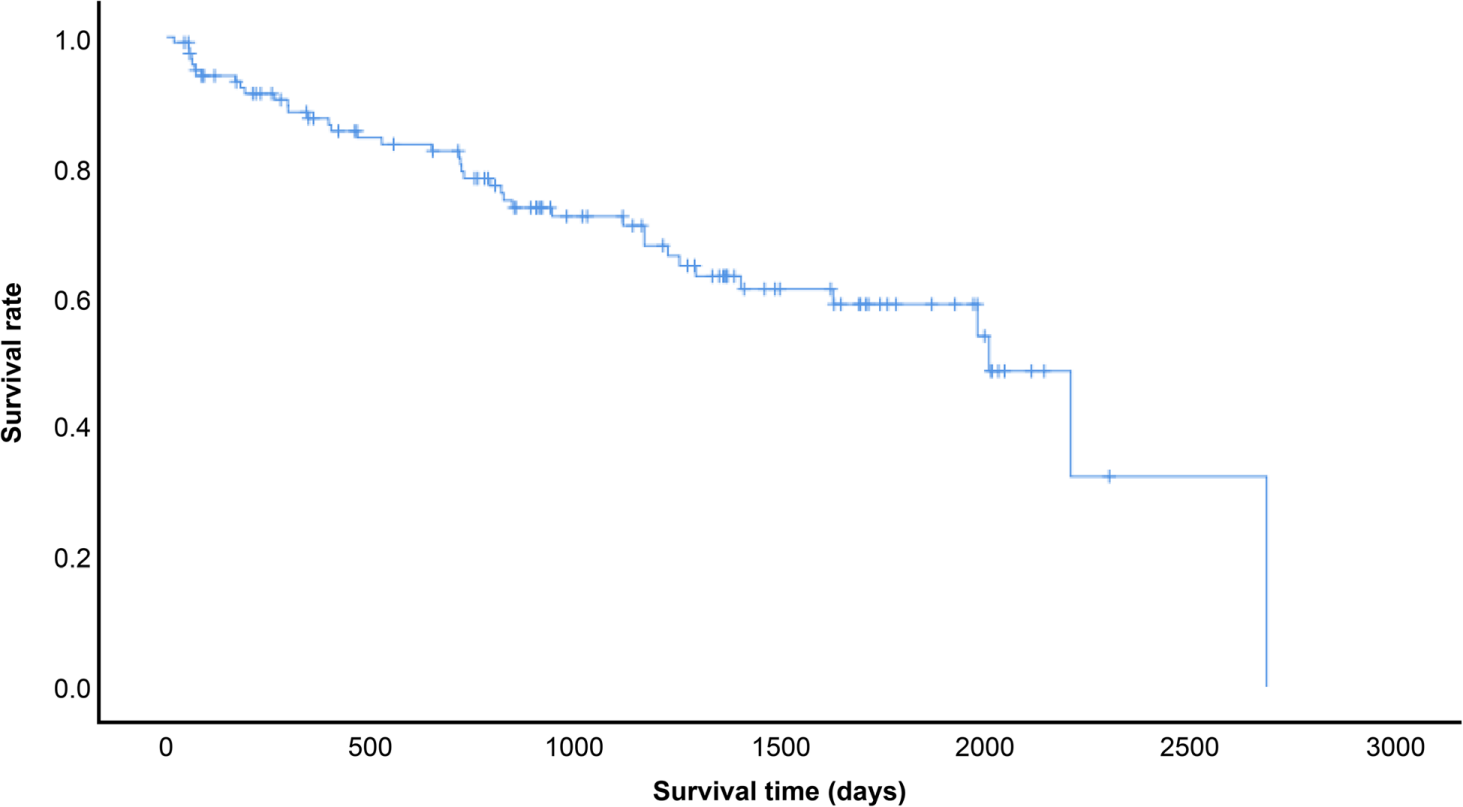
Survival curve for all 120 patients with non–small-cell lung cancer associated with interstitial pneumonia. The median survival time was 2011 days, and the one-year, three-year, and five-year survival rates were 87.5, 72.4, and 58.9%, respectively

Figure 5 shows the Kaplan–Meier survival curve for all 120 patients. The median survival time was 2011 days. The one-, three-, and five-year survival rates were 87.5, 72.4, and 58.9%, respectively. The causes of death were IP in 14 (12%) of the patients, lung cancer in 14 (12%), and other in 10 (8%).

The clinical outcomes for each pathological stage of cancer are shown in Table 4. Among the 46 patients with stage IA cancer, four (9%) experienced recurrence; the five-year survival rate for this stage was 69%.

**Table 4:** Outcomes for each pathological stage of lung cancer

	All stages (N = 120)	Stage Ia (***n*** = 46)	Stage Ib (***n*** = 27)	Stage IIa (***n*** = 16)	Stage IIb (***n*** = 5)	Stage III (***n*** = 10)	Stage IV (***n*** = 2)	Undetermined (***n*** = 14)
**Recurrence**	29 (24.2%)	4 (8.7%)	8 (29.6%)	8 (50.0%)	4 (80.0%)	3 (30.0%)	–	2 (14.3%)
**Induction of chemotherapy after recurrence**	10 (34.5%)	1 (25.0%)	2 (25.0%)	4 (50.0%)	0 (0%)	2 (66.7%)	–	1 (50.0%)
**Median survival time (days)**	2011	2211	Not reached	1116	818	1404	347	2011
**1-year survival rate**	87.5%	91.0%	88.0%	93.8%	75.0%	77.8%	50.0%	83.3%
**3-year survival arate**	72.4%	85.9%	74.5%	51.1%	–	64.8%	–	–
**5-year survival rate**	58.9%	69.2%	68.8%	22.7%	–	–	–	–

### Radiological results

Table 5 compares the clinical and pathological findings between groups A, B, and C (as defined in the Methods section). Of the 128 lung cancers, 89 (70%) were classified as group A, 29 (23%) as group B, and 10 (8%) as group C. There was a significant difference between the groups in patients with UIP pattern (*p* = 0.002) and the proportion of IPF patients (*p* = 0.014). The UIP pattern was exhibited by 49 (55%) patients in group A but by none in group C. All the patients in group B had pulmonary emphysema, but only 25% of the patients in group C had pulmonary emphysema (*p* < 0.001). There was also a significant difference between the groups in the histopathological type of cancer, specifically the proportion that were adenocarcinoma (*p* = 0.01).

**Table 5:** Clinical and pathological findings for the three groups defined in this study

	Characteristic	Group A (*n* = 89)	Group B (*n* = 29)	Group C (*n* = 10)	***P***
**HRCT IP pattern**	**UIP /Non-UIP**	49/40	11/18	0/10	**0.002**
**Clinical diagnosis of IPF**	**IPF/Non-IPF**	41/48	10/19	0/10	**0.014**
**Pulmonary emphysema**	**Yes/No**	69/20	29/0	2/8	**< 0.001**
**Postoperative acute exacerbation of IP**		7	1	0	N.S.
**Location of lung cancer**	**Upper lobe/Middle lobe/Lower lobe**	11/3/75	26/1/2	3/4/3	**< 0.001**
**Histopathology**	**Adenocarcinoma**	29	9	8	**0.01**
	**Adenosquamous carcinoma**	4	3	0	N.S.
	**Squamous cell carcinoma**	49	16	2	N.S.
	**Other**	7	1	0	N.S.
**EGFR mutation**	**Positive**	2	0	2	**0.047**

*Group A* cancer associated with fibrotic cysts, *group B* cancer associated with emphysematous tissue, *group C* cancer associated with normal lung tissue, *IP* interstitial pneumonia, *HRCT* high-resolution computed tomography, *UIP* usual interstitial pneumonia, *IPF* idiopathic pulmonary fibrosis, *N.S*. not significant, *EGFR* epidermal growth factor receptor

Group A included 29 (33%) adenocarcinomas and 49 (55%) squamous cell carcinomas and group B included 9 (31%) adenocarcinomas and 16 (55%) squamous cell carcinomas, whereas group C included 8 (80%) adenocarcinomas and 2 (20%) squamous cell carcinomas. EGFR mutations were most frequently detected in the patients in group C (*p* = 0.047). A further significant difference was observed in the location of the cancers (*p* < 0.001). In group A, 84% of the cancers were in the lower lobe; in group B, 90% of the cancers were in the upper lobe; and in group C, the cancers were located fairly evenly among the upper, middle, and lower lobes. Seven of the eight postoperative AE of IP cases were observed in group A.

### Pathological results

TPS levels of PD-L1 for 68 lung cancers in 64 patients were scored into three levels. In all cases, the independent classifications by the pathologist and the pulmonologist were identical. The numbers of patients with TPS ≥50, 1–49%, and 0% were 13 (19%), 40 (59%), and 15 (22%), respectively.

Table 6 summarizes the analysis of characteristics according to PD-L1 levels. The histological type of cancer differed between the TPS classifications: 11/15 (73%) of the cancers with TPS of < 1% were adenocarcinomas, whereas 20/40 (25%) of the cancers with TPS 1–49% were adenocarcinomas (*p* = 0.004). Only 2/15 (13%) of the cancers with TPS of < 1% were squamous cell carcinomas, whereas 24/40 (60%) of the cancers with TPS 1–49% were squamous cell carcinomas(*p* = 0.004). None of the patients with TPS of < 1% were classified into group B, whereas 12/40 (30%) and 4/13 (31%) of the cancers with TPS 1–49% and TPS of > 50% were classified into group B (*p* = 0.03). All postoperative AEs of IP were developed in the patients with TPS of < 1% (*p* = 0.015).

**Table 6:** Characteristics of the patients according to tumor proportion score for PD-L1

	PD-L1 < 1% (***n*** = 15)	PD-L1 1–49% (***n*** = 40)	PD-L1 ≥ 50% (***n*** = 13)	***P***
**HRCT IP pattern: UIP**	6	18	4	N.S.
**Emphysema**	13	31	12	N.S.
**Location of the cancer**				
**Upper lobe**	3	17	3	N.S.
**Middle lobe**	2	1	1	N.S.
**Lower lobe**	10	22	9	N.S.
**Group A**	13	26	8	N.S.
**group B**	0	12	4	0.03
**group C**	2	2	1	N.S.
**Pathological findings**				
**Adenocarcinoma**	11	10	5	0.004
**Squamous cell carcinoma**	2	25	6	0.004
**Other**	2	5	2	N.S.
**EGFR mutation positive**	2	0	1	N.S.
**Postoperative acute exacerbation of IP**	3	0	0	0.015

*IP* interstitial pneumonia, *HRCT* high-resolution computed tomography, *UIP* usual interstitial pneumonia, *IPF* idiopathic pulmonary fibrosis, *N.S.* not significant, *EGFR* epidermal growth factor receptor, *PD-L1* programmed cell death ligand 1, *group A* cancer associated with fibrotic cysts, *group B* cancer associated with emphysematous tissue, *group C* cancer associated with normal lung tissue

## Discussion

This study retrospectively investigated clinical, radiological, and pathological characteristics of 120 consecutive patients with NSCLC associated with IP who underwent surgery. Eight (7%) of the patients experienced postoperative AE of IP, with three of these (38%) dying. The patients who developed AE tended to have undergone lobectomy and to have lower %VC. The five-year survival rate of the 46 patients at pathological stage IA was 69%, with only four (9%) experiencing a recurrence of the lung cancer. We classified the lung cancer with IP into three groups according to the location where the cancer developed by HRCT. Each group showed characteristic features in terms of tumor location, histopathology, PD-L1 expression, and postoperative AE frequency. To our knowledge, this study is the first to classify lung cancers with IP according to the location of cancer development. Our study findings may help pulmonologists manage patients with resectable lung cancers accompanied with IP.

Studies have reported postoperative AE of IP in patients with NSCLC [8, 16–18]. In the largest of these [8], 9.3% of patients experienced postoperative AE, with a mortality rate of 43.9%. In comparison, our results of 7% with 38% mortality were marginally better. The relatively low proportion of patients with the UIP pattern in the present study may have influenced this result because the UIP pattern is thought to be a risk factor for developing AE of IP [8, 32]. In addition, almost all our patients underwent VATS, not open surgery. Although the safety and efficacy of VATS for patients with IP have not yet been established, the high proportion of patients who underwent VATS may have contributed to our good result [15]. Sato et al. proposed a scoring system to predict postoperative AE of IP [8, 33], based on a history of AE of IP, the surgical procedure, the UIP pattern, male sex, the preoperative use of steroids, elevated serum Krebs von den Lungen-6 level, and low %VC. In the present study, the patients who developed AE of IP tended to have undergone lobectomy and to have lower %VC; however, %VC and lobectomy were the only predictors for AE of IP in this study. This may partially be explained by the small study sample size and partially by the low occurrence rate of AE of IP.

We also analyzed postoperative treatment and long-term outcomes. When treating patients with advanced or recurrent NSCLC and IP, consideration should be given to the possible AE of preexisting IP by cancer treatments such as chemotherapy or immunotherapy. In the present study, the most frequently chosen option for the patients who experienced recurrence was best supportive care because of concerns about the AE of IP as a result of chemotherapy [9, 10]. Of the 46 patients with stage IA cancer, four (9%) experienced recurrence; this rate is comparable to that of patients without IP [34–38]. However, the five-year survival rate of the patients with stage I cancer was only 69%. Sato et al. also reported a low 5-year survival rate in 59% of patients with stage IA NSCLC [39]. These might be explained by the high proportion of patients who received only best supportive care following the recurrence or by the high mortality rate of IP itself. Notably, three patients with stage Ia NSCLC developed postoperative AE of IP. Almost half of the deaths of the patients with NSCLC and IP were owing to IP; thus, the extremely high mortality rate of IP should be considered when developing strategies for these patients.

We classified the lung cancers with IP into three groups, which each showed characteristic features. Group A included cancers located under fibrotic shadows. Group A tended to have squamous cell carcinoma and showed the UIP pattern. Lung cancers were mainly located in the lower lobes in this group. Most cases of postoperative AE of IP were observed in this group. Extremely high attention should be paid to AE of IP when treating patients in this group. Furthermore, some patients with secondary IP and those without UIP were included in group A. Thus, fibrosis leads to lung cancer development regardless of the etiology or radiological pattern of IP. Group C included cancers of normal lung. None of the patients in group C showed the UIP pattern and most of the cancers were adenocarcinoma. The frequency of epidermal growth factor receptor mutation-positive lung cancer was highest in this group. We speculate that the cancers in this group developed independently from the underlying IP. Driver mutations should always be assessed in this group. Group B included cancers of or near emphysematous tissue. The characteristics of this group were similar to those reported in a previous study of lung cancer associated with emphysema [40]. We speculate that the cancers in this group developed in association with the emphysema. No patients were PD-L1 negative; thus, anti-programmed death-1 or PD-L1 drugs could be used for this group.

Lung cancers in the upper or middle lobes in group A and those in the middle or lower lobes in group B were minority cases (only 17 cases). Among the former 14 cases in group A whose lung cancers developed in the upper or middle lobes, eight had a non-UIP pattern of IP. However, 13 patients had lower-lobe–predominant fibrosis, whereas only one patient had upper-lobe–predominant fibrosis. Only three patients in group B had lung cancers in their middle or lower lobes. Pulmonary emphysema was detected not only in the upper lobes but also in the middle or lower lobes, and lung cancers developed near the emphysema in the middle or lower lobes. The local radiological findings between these minor cases and the rest of the cases had no difference. Hence, fibrosis or emphysema itself can cause lung cancer development irrespective of the location or lobes.

PD-L1 expression in lung cancers was also assessed in this study. The proportion of PD-L1 expression-positive patients was comparable to those reported previously studies [26, 27]. Most cancers with PD-L1 negative were adenocarcinomas, and no patient with PD-L1 negative was classified into group B. All postoperative AE of IP developed in patients with TPS of < 1%. However, we cannot understand the significance of these results owing to the small cohort size. Further studies are needed to understand the mechanism underlying PD-L1 expression.

This study had some strengths; it was one of the largest retrospective studies of NSCLC with IP conducted by a single institution, and it considered long-term outcomes as well as detailed analyses of these patients. However, the study had several limitations. It was a retrospective and single-center study with a relatively small sample size. We did not directly compare patients with IP to patients without IP. A larger, multicenter analysis should be performed to establish clear predictors for the development of postoperative AE of IP.

## Conclusions

We assessed 120 consecutive lung cancer patients with IP and classified them into three groups according to the location where the cancer developed. Each group had characteristic features in terms of tumor location, histopathology, PD-L1 expression, and frequency of postoperative AE. These findings should help inform the treatment of lung cancers associated with IP.

## Data Availability

All data generated or analyzed during this study are included in this published article.

## Abbreviations

AE: Acute exacerbation
GAP: Gender–Age–Physiology index
HRCT: High-resolution computed tomography
IP: Interstitial pneumonia
IPF: Idiopathic pulmonary fibrosis
NSCLC: Non–small cell lung cancer
PD-L1: Programmed cell death ligand 1
TPS: Tumor proportion score
UIP: Usual interstitial pneumonia pattern
%VC: Percent predicted vital capacity
